# Social Eating Among Child and Adult Hospital Patients: A Scoping Review

**DOI:** 10.3390/ijerph22050796

**Published:** 2025-05-18

**Authors:** Emily Barnes, Rebecca O’Connell, Claire Thompson, Jessica Brock, Caroline Heyes, Nancy Bostock

**Affiliations:** 1 Centre for Research in Public Health and Community Care (CRIPACC), School of Health, Medicine and Life Sciences, University of Hertfordshire, Hatfield AL10 9AB, UK; eb23aaq@herts.ac.uk (E.B.); c.thompson25@herts.ac.uk (C.T.); 2 Centre for Food Policy, City St George’s, University of London, Northampton Square, London EC1V 0HB, UK; jessica.brock.2@citystgeorges.ac.uk; 3 Department of Nutrition and Dietetics, Cambridge University Hospitals, Hills Rd, Cambridge CB2 0QQ, UK; c.heyes@nhs.net; 4 Cambridgeshire & Peterborough NHS Foundation Trust, Elizabeth House, Fulbourn, Cambridge CB21 5EF, UK; nancy.bostock@cpft.nhs.uk

**Keywords:** social eating, commensality, hospital food, scoping review, nutrition

## Abstract

Current policy attention to the ‘public plate’ includes a focus on food in hospitals. Recommendations for much-needed improvements include the provision of opportunities for patients to engage in ‘social dining’, which has been shown to have a range of benefits for health and well-being. However, there has been no synthesis of the literature on the topic to date. This scoping review sets out to identify and examine different types of evidence on social dining in the hospital context, factors shaping its implementation and impact, and knowledge gaps. Following comprehensive searches of two databases and a thorough screening process, 38 papers were included in the review. The outcomes and impacts of social dining were measured in a variety of ways across the studies. Social dining in hospitals can impact dietary intake and nutritional outcomes and have implications for patient well-being. These effects are moderated by a range of factors, including the age and needs of the patient group, eating environment, and presence of staff and visitors. Future research needs to focus on children and their families, an under-researched patient group that may be especially likely to benefit from the opportunity to eat with others in the hospital.

## 1. Introduction

Internationally, and in the UK, there is growing policy attention to the importance of food in institutional settings, including hospitals [1,2]. Within recent public inquiries into food and eating in hospitals, ‘social dining’ emerges as an important theme, with recommendations made for hospitals to explore the potential that eating with others has on improving food intake, patient experience, and outcomes [3,4,5]. For example, the most recent UK Inquiry, the Independent Review of National Health Service (NHS) Hospital Food [3] suggests that ‘it is important that […] social aspects of the dining experience are considered’ and that at mealtimes, ‘the presence of friends, family or volunteers can be beneficial to a patient’s dining experience’ and to ‘help patients to eat more than they would otherwise’ (p. 38). The review notes that around two-thirds of patients surveyed by the Patients Association said they ‘might’ or would ‘definitely’ like to use a shared dining space if it is available, although the sample size was small. However, whilst there is a growing body of research on some aspects of the eating experience in hospital, such as the impact of protected (uninterrupted) mealtimes on malnutrition [6], feelings of loneliness and quality of life [5] there has as yet been no synthesis of the evidence to support recommendations on social dining in hospitals, or to inform their implementation. This is particularly the case for children and young people, for whom there is a lack of research on hospital food and eating more generally.

Eating, beyond being a biological necessity, is a central tenet of our social lives [7]. In sociological and anthropological approaches to food and eating, meals are understood to be ‘social’ events, in that they are socially constructed, socially organised and fulfil social functions [8]. The question of who eats with whom, or ‘commensality’, has been a key concern in the social scientific analysis of eating occasions [9]. In the field of public health, research has also examined the impact of eating with others, or eating alone, on nutritional intake and social and psychosocial outcomes, particularly among older people [10].

With respect to children and young people (CYP), studies exploring their food practices, experiences and nutritional intake have mostly been carried out in the private sphere of the family home, or in educational settings, which are central spaces in most children’s lives [11] (for an exception, exploring children’s food practices in residential care, see Dorrer et al. [12]). Yet research exploring CYP’s food and eating practices outside of home and school settings, or in other institutional spaces, such as hospitals, remains underdeveloped. For many children and young people who are hospitalised, the sudden loss of sociality at school plays a large role in negative inpatient experiences [13,14,15,16]. In other population groups, such as older adults, patients often report experiencing feelings of loneliness while they are in a hospital setting [17,18], and, in some cases, communal eating programmes have been specifically designed to mitigate such feelings of isolation and to improve perceived quality of life [5]. However, we are unaware of any previous attempts to synthesise evidence on this topic.

This review aims to build upon the growing body of research exploring the role of food and eating in hospital care by exploring food practices in these settings from a social perspective. It examines what evidence there is regarding social eating in the context of a hospital ward, what factors are important to consider regarding implementation and impacts, and what the included research says about how social eating can be supported. It also reflects upon how the studies it includes might be relevant to the more specific population group of children and young people in hospitals, highlighting a lack of research in this area.

Preliminary searches of the literature conducted on Google Scholar and PubMed using varying sets of initial search terms identified the lack of research in this area, and that the little evidence found spanned a range of disciplines and methodological approaches. Some population groups, particularly children and young people, were also underrepresented in the retrieved results. These initial searches also helped identify further search terms and phrases relevant to the body of literature that would inform the final search strategy (i.e., from reviewing keywords). A scoping review was therefore selected as an appropriate approach, since, as noted by Arksey and O’Malley [19], scoping reviews can address topics or questions that are broader than those dealt with in systematic reviews and allow for variation in study design or methodology. Additionally, this type of review lends itself to the identification of evidence gaps [ibid].

## 2. Materials and Methods

This scoping review set out to answer the following questions:

- What evidence exists regarding social eating in hospitals, more specifically in hospital wards?
- What factors are important to consider when exploring the implementation and potential impacts of social eating in a hospital context?
- What does the identified research tell us about how social eating in hospitals can be supported, especially in the context of children’s hospitalisation?

### 2.1. Eligibility Criteria

The inclusion or eligibility criteria provided the basis for decisions on sources to be included in the review. There are a range of mnemonics for different types of review and research questions. We have employed the “PCC” mnemonic, which stands for Population, Concept, and Context, to structure our review [20].

### 2.2. Population

Preliminary searches focusing only on social eating among the population group of CYP in hospitals yielded too few results. It was thus decided that the review would include older participants in order to fully explore its aims and provide greater breadth on the subject. Therefore, no restrictions were placed on participant age, but extra search terms specific to the population of CYP were added to ensure that any results focusing on this group were captured. Since a range of actors are involved in the provision of meals in hospital settings, this review also included studies with participants other than patients (such as health-care professionals, volunteers, and patient families) in order to offer a holistic representation of the topic.

### 2.3. Context

Context was understood in this review as referring to the setting in which the research took place. Research was included in this review if it was based on experiences, perspectives, or outcomes reported in a hospital, clinical, or medical care inpatient setting. Works carried out in other contexts, such as nursing or care homes, were excluded, and papers involving patients who had been discharged or who no longer used hospital services were also excluded.

### 2.4. Concept

This review uses the terms ‘social eating’ and ‘social dining’ interchangeably with the word ‘commensality’, often employed by the social sciences to refer broadly to the act of eating together with other people [21]. In the context of a hospital, eating together might be among patients as well as between patients and visitors or staff. Shared meals necessarily entail coordination or synchronisation of people’s schedules, foods, or both [8,22]. However, there is much debate over the definition of commensality as well as its outcomes and impacts [23]. For example, whilst shared family meals are widely encouraged for health and other reasons, it has been shown for children that eating the same foods as their parents has a greater impact on nutritional outcomes than other mealtime variables, such as eating at the same time [24]. However, studies interested in alleviating loneliness among older adults have found that shared mealtimes, such as luncheon clubs, make a significant difference to psychosocial outcomes [10]. In the context of a larger institution, such as a hospital, for example, variety plays an important part in catering to the needs of different cultures, conditions, and preferences [25], and the synchronisation of tastes and foods is unlikely to be recommended or feasible. Furthermore, this review aims to identify and synthesise a variety of outcome measures such as levels of interaction among inpatients, intake, and attitudes towards eating commensally. Papers exclusively exploring eating assistance (without participants eating together) in hospitals were excluded, as there is already a large body of literature researching this topic, and they did not necessarily align with this review’s definition of social eating. Research that explored both shared/social dining experiences and solitary dining was included, as solitary dining provided a basis for comparison, such that a broader understanding of eating in hospitals could be gained.

Reporting guidelines outline a minimum set of items to include in research reports and have been shown to increase methodological transparency and uptake of research findings [26]. This review followed the PRISMA-ScR (Preferred Reporting Items for Systematic Reviews and Meta-Analyses—extension for Scoping Reviews) reporting guidance set out by Tricco et al. [26] and situates its findings within the broader implications for mealtimes in a paediatric ward. Systematic and scoping reviews were excluded from this review.

### 2.5. Search Strategy

Arksey and O’Malley’s [19] methodological framework for scoping reviews was used as the basis for this research, in which: the research question and relevant studies were identified; studies were selected to be included in the review; the data extracted from these included studies were charted in Table A1 collated and summarised. Two electronic databases were used to carry out the search: PubMed and Scopus, as they provide a comprehensive collection of works with a focus on public health and social science while capturing results from a range of theoretical perspectives and study designs. Search terms and the inclusion/exclusion criteria for this review were developed using the PCC format as recommended by Pollock et al. [20]. The search terms used to carry out the database searches can be found in Table A2. Searches were carried out at the title, abstract, and keyword levels, and search strategies were adapted for the two databases where they differed, for example, where they used different proximity operators. No limitations were placed on publication year due to the lack of research yielded in the preliminary searches.

### 2.6. Article Selection

The searches were carried out in February 2024, and the screening software, Rayyan, was used to assess the results against predefined eligibility criteria, following recommendations of Mak and Thomas [27]. A 5% (*n* = 77) subset of the results was subject to a successful initial, independent, blind screening at the title and abstract levels by four reviewers to assess the rigidity of the inclusion and exclusion criteria before the formal screening process took place. The complete set of papers captured by the database searches was initially screened at a title and abstract level, and irrelevant papers were excluded. The full texts of the remaining papers were then read, and an inclusion/exclusion decision was made based on the eligibility criteria. Further relevant works that were not captured by the database search were identified from the reference lists of included studies when read at full text to ensure the review comprehensively reflected the available literature.

## 3. Results

**Figure 1:**
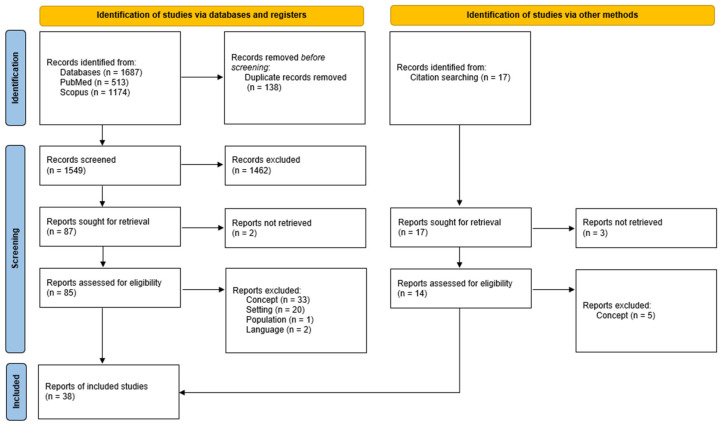
PRISMA Flow Diagram, documenting database searches, the number of records screened, and the number of full texts retrieved.

In total, as shown in Figure 1, 1687 papers were retrieved from the two electronic databases searched. Of these, 138 were identified as duplicates and removed, leaving 1549 papers to be screened. After the title, abstract, and keyword screening was carried out, 1462 were found not to be relevant. The remaining 87 papers were screened at full text, and 29 met the predefined and tested eligibility criteria. Any papers that the first author needed further consultation on were screened at full text by a second reviewer. A further nine texts were found via citation searching carried out from these works, meaning that 38 papers were included in the final selection. Most studies were excluded as they did not meet the inclusion criteria for the concept of social eating, which this review defines as eating with others. Other reasons for exclusion included focusing on discharged patients’ experiences of mealtimes outside of the hospital, or research taking place in settings such as residential nursing homes. The relevant data from each article were then extracted and charted by one reviewer.

### 3.1. Study Characteristics—Date, Location, and Setting

The studies meeting the inclusion criteria were all published between 1980 and 2024, with most of the studies being published in the last 15 years and nearly 20 percent of the papers being published in 2021 (7/38). From the 38 included papers, the research was carried out in nine countries, with most being carried out in the UK (10), Denmark (9), Sweden (6) and Australia (6) and the others based in Norway (2), Canada (2), Belgium (1), Singapore (1) and the US (1). The studies were based on a variety of inpatient settings, including rehabilitation units, neurological care units, and paediatric care facilities. Some studies were carried out across multiple wards or hospital locations. Four studies were conducted in residential eating disorder units.

Mealtime locations varied from communal dining rooms to eating at the bedside or in bed. Some studies compared the experiences or outcomes of different eating locations [28,29,30,31], while others used control groups to explore the impact of environmental interventions in the dining rooms or the use of dining rooms in general [32,33].

### 3.2. Study Characteristics—Study Design

Twenty-three of the studies included in this review used qualitative methods to collect data, eight used quantitative methods, and seven employed a mixed-method approach. Of the qualitative studies, most employed participant observation and semi-structured interviews. Other qualitative methodologies included focus groups and visual (photography-based) research. The quantitative studies measured the impact of social dining on a range of outcomes through methods such as plate weighing, counting the number of social interactions during mealtimes, and via surveys with scalable answers. Two studies conducted on eating disorder wards used an established research instrument to record the number of eating disorder behaviours that occurred during group mealtimes. The mixed methods studies used a combination of these methods, as well as other data collection tools, such as questionnaires.

Thirteen papers used interventions in their study designs. Interventions included introducing communal dining to the ward. Of these interventional studies, four used control groups [29,30,31]. Other changes introduced included environmental enrichment such as moving furniture, adding tablecloths or cutlery on the dining tables [32,33,34,35], or the introduction of background music to mealtimes [36,37]. In two papers reporting the same study [38,39], an intervention promoting a quieter and calmer atmosphere (modelled on the ‘protected mealtimes’ scheme) was introduced. Two papers, written by Gardner and Trueman and Gardner et al., were based on an intervention study on an eating disorder ward in which staff received further mealtime training, and a ‘host’ role was introduced to the dining room [40,41].

### 3.3. Study Characteristics—Participants

This review makes a distinction between the patient group(s) in the wards where research was being conducted and the individuals the studies involved as participants, as not all studies involved patients as primary participants.

Half (19/38) of the studies took place in wards catering to patients over 60 years old or described as ‘geriatric’. Fourteen were based in wards with patients of a wide range of ages, with two of these participants being aged under and over 18 years old. Only three of the studies included in this review were based on research carried out in paediatric wards with children and their families. Two of the papers did not explicitly refer to the ages of the patients involved. Four of the studies included in this review were based in settings dedicated to caring for child and adult patients with eating disorders.

**Figure 2:**
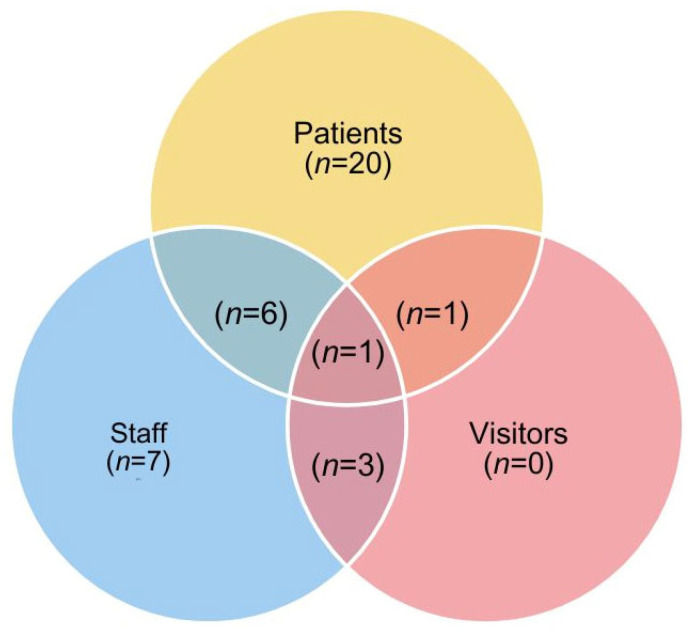
The number of studies using the different participant groups [42].

Qualitative studies in this review included the views and experiences of multiple participant groups involved in hospital mealtime care aside from patients, such as hospital staff or patients’ families, to develop a holistic understanding of the strengths and challenges of dining with others in the hospital. Figure 2 charts the information relating to participant groups across the included studies. Twenty studies only included patients as participants in their research, six included both patients and staff as participants, and seven focused only on the perspectives of staff. Three papers included both staff and patients’ visitors in their study, and one explored the perspectives of both patients and their visitors. Only one paper involved patients, visitors, and staff in their study.

Study sample sizes ranged from fewer than ten to almost three hundred participants. Four studies included <10 participants, eight studies included between eleven and 20 participants, ten studies had a sample size of between 21 and 30 participants, seven had between 30 and 60 participants, one study had a sample size of 84 participants and two studies had samples sizes of >100, with 149 and 296 participants. Six studies made no explicit reference to the number of participants who took part.

### 3.4. Study Characteristics—Findings

The results of the included studies have been synthesised and structured according to shared themes or findings that arose from across the literature, relating to the multiple dimensions and practical considerations of social dining in hospitals that should be considered. For example, the biological implications of eating together that arise from results relating to intake, patient preference for dining location, the experiences of eating with other hospital patients, the practical role of staff and emotional impact of eating with family and friends and the role the physical environment played in facilitating or interfering with opportunities for sociality. The selection of these themes not only allowed this review to broadly synthesise the results of studies with varying designs and focus, but also provided a holistic and multidimensional account of social dining in a hospital setting.

#### 3.4.1. Measuring Sociality

In this review, defining social eating as simply ‘eating with others’ means that the nature and level of ‘sociality’ were characterised in a range of ways across the studies included. Three out of the eight quantitative studies measured patient sociality during mealtimes through the number of instances of interaction among patients [33,34,43]. However, Melin and Götestam’s quantitative study with psychogeriatric patients based in Sweden also included interactions between patients and staff who were not dining themselves [33]. In other cases, the social setting of the dining room facilitated only very minimal social interactions [44], and patients were sometimes even silent during mealtimes [28]. In other cases, small talk was observed [38], and more in-depth conversations were seen as an indispensable part of the dining experience [37]. One study even explored the content and mood of conversations between patients before and after an intervention [37].

#### 3.4.2. Reported Impact of Aspects of Social Dining on Dietary Intake

Among the included studies, six measured the impact of changes to the dining room mealtime setting on dietary intake. Of these, five reported a significant increase in dietary intake. Edwards and Hartwell’s quantitative study examined adult patient nutrient intake when eating at different locations [31], comparing a group eating around a table to two control groups, the first ate sitting next to their bed, and the second ate while sitting in bed. The results showed an increase in the mean daily energy intake for the dining table group over the other two, and the intake levels of carbohydrates, fats, and protein were greater for the dining table group during lunchtime. Wright et al.’s quantitative study with older adults also used a control group to measure intake against a group encouraged to use a ward dining room and showed that those in the dining room group had significantly higher intakes of energy than the control group (mean intake of 489 kcal compared to 360 kcal) [30]. It should be noted that the results provided by Edwards and Hartwell and Wright et al. were derived from small samples, as Edwards and Hartwell’s study only included 13 participants and Wright et al.’s work was based on findings from patients who visited the dining room four times on average [30,31].

In Markovski et al.’s quantitative study comparing intake at different mealtime locations [29], energy and protein intake increased (by 20% or more) when older patients with significant cognitive impairment ate in the dining room, compared to the bedside. Holst et al.’s mixed methods study reported that environmental enrichment in an adult ward, including background music, led to increases in energy intake [36], though the same was not seen for protein intake. Paquet et al.’s quantitative study measuring older (65<) patient interactions during mealtimes reported a positive relationship between the total number of communal interactions or behaviours of individuals and their energy intake consumed during that meal [43]. Walton et al.’s mixed methods study exploring the various influences on older adult dietary intake found that ‘a social approach’ to mealtimes, along with other strategies used by a private hospital in the study [45], was conducive to increased dietary intake, however, no concrete data are provided to support this account. The exception that did not report a significant change in dietary intake was Mathiesen et al.’s mixed methods study, which introduced music to mealtimes in the dining room of a specialist brain injury ward [37]. However, average fluid intake increased.

#### 3.4.3. Patient Preference for Dining Location

While not a key concern in the reviewed studies, around a quarter (9/38) referenced patient preferences regarding dining location. In Markovski et al.’s quantitative study the majority of the adult patients included in the study (68%) stated that the dining room was their preferred eating site [29], and Walton et al. note that although the primary eating location was the bedside for the older patient participants in their research [45], the dining room was popular for those who were mobile at lunch and in the evening. Sidenvall et al. note that all of the older adult patients with moderate eating problems involved in their qualitative study said that they wanted to eat in the dining room, although one patient in their study was reluctant [42], and those with more severe physical conditions found it difficult to eat in the dining room and preferred the freedom provided by the privacy of their room. Similarly, Mårtensson et al.’s qualitative work with paediatric oncology patients using a gastrostomy tube also found that difficulties experienced by the patients eating orally meant they did not always want to eat at a table with others [46]. On the other hand, in Sundal’s qualitative study [47], one of the child patients on a general paediatric ward expressed a desire to eat at a table instead of in their bed. Baptiste et al. reported that half (4 out of 8) of the older adult participants involved in their qualitative study said that they preferred eating in the dining room [28], two said they did not have a preference, and the remaining two preferred eating in their rooms. In Beck et al.’s qualitative study involving adults aged 27–78 years [48], many of the patients chose to eat in bed because it provided more privacy than the communal dining option, in which dining tables were located in the ward hallway. Sidenvall notes in their qualitative research on older adults that staff did not ask patients about their preference for eating location [49]. In Bryon et al.’s qualitative study [50], hospital staff understood that their geriatric patients would prefer eating alone at times, but providing an individualised meal service would make it harder for staff to manage. Baptiste et al. also note that some older adult patients reported having limited control over their dining location for lunch and dinner [28], which was mostly determined by staff.

#### 3.4.4. Influence of ‘Tablemates’

Dining with other patients was found to have different effects on eating across different studies. The behaviour of ‘table-mates’, a term adopted by Sidenvall to refer to other people eating at the same table or in the vicinity, was found to have a negative influence on the eating experience in four studies [51]. Papers written by Sidenvall et al., and Sidenvall report that the ‘unpleasant behavior’ of others at the dining table (for example, using personal cutlery to serve food) made some patients in a rehabilitation and long-term care clinic experience discomfort [42,49,51]

Long et al.’s paper noted that, in the context of an eating disorder unit, patients could also influence each other’s eating habits negatively, and rivalries or competitions over food [52]—such as trying to be the last to finish their meal—could develop when eating together. Beck et al. (2018) argue that the varying conditions of the patients present during mealtimes made socialisation difficult and eating unappetizing [48]. In Furness et al.’s research [53], ‘other patients’ were identified as a factor inhibiting meal intake and seen as a deterrent to the mealtime environment. Conversely, ten papers explored the difficult experiences of patients who, as a result of their condition or treatment, found eating with others uncomfortable due to feelings of shame they felt as a result of their impaired eating abilities [28,35,42,46,48,49,51,52,54,55]. Hartwell et al.’s study [35], for example, noted that patients relying on equipment, such as catheters and drips, felt that their presence in the social dining space was inappropriate and undignified. Sidenvall et al.’s research observed that patients in pain, or who had difficulty eating [51], preferred to concentrate on their plate, rather than making conversation with others. Bryon et al.’s qualitative study of older adults eating in a common dining room reported that those with special diets ate in a different location [50], as historically, patients had traded meals or had taken things from the plates of others.

On the other hand, seven studies reported that eating with others in the hospital was regarded as providing a sense of community, togetherness, and affinity between patients [38,39,42,51,54,56,57]. Hartwell et al.’s paper noted that in many cases [35], staff reported that patients eating with other patients facilitated a more dignified hospital experience, while Bryon et al. suggest that the collective responsibilities of the older patients in creating a positive communal mealtime experience [50]—such as laying the table—provided them with a sense of agency. Beck et al.’s study observed that the sense of community experienced during mealtimes was compared by patients to dining situations outside of the hospital [57], such as eating lunch with colleagues at work.

#### 3.4.5. Staff and Visitor Involvement in Communal Dining

Staff were reported as having varying roles during communal mealtimes. In some cases, staff ate or had historically eaten with patients in the dining room [40,41,44,58]. Three studies [40,41,59] two of which were mixed methods studies carried out in eating disorder units and the other a qualitative study involving adult patients, explored staff involvement through the ‘host’ role. This was reported to be beneficial in reducing behaviours related to disordered eating, and one suggested it was successful in promoting a sense of commensality while patients ate together [59]. In Dickinson et al.’s qualitative study of older adults, the staff facilitation of communal meals was perceived as poor by patients [44]; despite this, all participants interviewed in the study said they would like the opportunity to share meals with staff from their unit. Jong et al.’s qualitative study noted that encouragement from staff for the older patients to utilise the communal dining area led to an awareness among patients about its perceived benefits [55]. In one instance, from the perspective of staff, communal dining environments were reported as reducing legwork for staff during mealtimes [35], though other studies noted that factors such as time pressures and transporting patients from room to room were perceived as making the delivery of meal services difficult [55]. The location of the dining room itself was also seen as important by staff involved in Jong et al.’s study [55], for example, its proximity to other facilities such as toilets.

Young et al.’s qualitative study of older inpatients reported that participants said the ideal mealtime scenario would involve welcoming families and caregivers [60]. Additionally, caregivers in Bryon et al.’s qualitative study recognised that their involvement had a significant impact on patient experiences of mealtimes, although not all volunteers were reported as eating with patients [50]. Ottrey et al.’s qualitative research on an adult subacute ward draws on examples in which visitors and patients were occasionally observed to create shared meal experiences [61].

In regards to involvement of patient families, staff participants in Sundal and Vatne’s qualitative paper noted that child patients’ parents played an important role in facilitating a sense of normality around mealtimes [62], while staff in Neo et al.’s study felt that family involvement in the mealtimes of older people was critical in providing adequate nutritional care [63].

#### 3.4.6. Eating Environment

Over half (22/39) of the studies reflected on the impact of the physical and/or sensory environment on the mealtime experience. Hartwell et al.’s quantitative study showed that, after the quality of the food and service, the social and eating environment were perceived as being important in predicting patient satisfaction [64], while Furness et al.’s mixed methods research identified sensory aspects, such as surrounding noises and their impact on ambience, accessibility and functionality of the dining space, as being important to patients when eating [53]. Furthermore, when aspects of the physical environment were considered poor, the possibility of social interaction seemed to be negatively impacted [44,55]. Material changes to the environment, labelled as environmental enrichment or enhancement in some studies, had a range of outcomes, including increased feelings of dignity or normality [35]. The layout of the dining room also arose as an important factor [52]. In one study, changes in the arrangement of furniture led to more opportunities for social interaction, while simultaneously creating a crowded feeling in the dining room [59].

Additions to the dining space such as tablecloths, flowers on the tables, or water jugs, were perceived by patients to convey a sense of care and familiarity [36,38], while other aspects of the physical environment, such as windows, made for a pleasant dining experience as reported by patients involved in one study [28]. In Mathiesen et al.’s mixed methods study [37], environmental interventions (in this case, the introduction of music) were found to shift the content of mealtime conversations between adult patients in the study, away from serious topics towards more lighthearted ones. In Justesen et al.’s qualitative research on gynecology and cardiology wards [65], patients transformed the physical environment of the ward into a ‘cafe’, which was perceived by them to be a space of hospitality. Both Sundal and Young et al’s qualitative studies reflected on the importance of ‘homeliness’ in the dining setting [47,60], for different age groups, with Young et al. involving older inpatients and Sundal focusing on the parents and families of paediatric patients [60].

Some included studies reflected on the impact of the clinical setting on mealtime enjoyment. For example, studies by Mathiesen et al. and Jonsson and Nyberg reported that the noises of hospital machinery were observed as not being conducive to sociality, and emphasized the importance of moments of silence or interrupted conversations [37,59], while adult patients in Larsen et al.’s qualitative study observed that the dining environment was reminiscent of illness and offered no separation between the hospital setting and the mealtime setting [54]. Hartwell et al.’s qualitative study [35], on the other hand, reported that eating in a designated dining area separate from the ward made for a more hygienic eating environment away from the clutter of the bedside.

## 4. Discussion

To our knowledge, this scoping review is unique in its synthesis of evidence on social eating in hospitals among various patient populations, including children and young people. Other similar reviews in this area, however, explore different contexts [66] population groups with specific conditions [67], frame social eating amongst other mealtime interventions [68] and focus on other related areas of interest such as ‘assisted eating’, where patients are accompanied at mealtimes but where eating with others does not necessarily take place [69]. In synthesising the results of research in this area thus far, this review presents the multiple social, physical, and emotional dimensions of eating with others in the hospital that should be considered. What this paper also draws on is the relationship between the physical environment and experiences of sociality in the context of food and eating, pointing towards a relational view of hospital spaces. Relational considerations of place have been applied in health geography and to food environments [70] but have yet to be explored in depth regarding institutional settings like hospitals.

The aim of this review was to explore existing academic research on commensality or ‘eating with others’ in hospitals, to reflect on factors important in its implementation and impacts, and how it might be supported. Defining social eating as simply eating with other people meant that a breadth of studies could be included; it also meant that there was diversity among the studies in how social eating was characterised and measured. Thirty-eight papers were included in this review, and all touched on social eating in some capacity, though the nature of sociality varied. In most cases, references to social eating came out of broader explorations of hospital care and mealtimes, and less than half (14/38) of the studies explicitly referred to social dining or communal behaviours or interactions at mealtimes when stating their aims. In the other studies reviewed, commensality emerged as a theme or outcome from the research and was not the primary focus. Most of the literature (33/39) was based on studies carried out with adult or older adult respondents or patients in a hospital environment catering towards conditions associated with older age. Few studies (8/39) were concerned with children or young people (CYP) with a mean age under 25. As we explore below, particular considerations arising in the care of children in paediatric settings mean that some findings are more or less relevant to apply to the context of a children’s hospital.

Overall, the studies included in this review provide a broad account of the advantages and disadvantages of social dining on hospital wards for different patient groups. One study makes recommendations for the use of a dining room [48], while the staff in Neo et al.’s study recommended that families and visitors actively take part in sharing mealtimes with patients [63]. Social mealtimes offered opportunities for patients to alleviate feelings of boredom or loneliness [28] and to develop a sense of togetherness [39]. Other studies, however, explored the difficulties in implementing social dining practices from the perspective of patients who did not always enjoy the communal dining experience due to issues arising from their conditions or treatment. Logistical factors also arose from interviews carried out with staff in two studies. On the one hand, the dining room meal service was seen as being more efficient as everything was in one location [38], while in other cases, issues such as time pressures and the levels of supervision required were exacerbated using this model [55].

One key factor that arose in the literature as crucial when reflecting on social mealtimes was the physical dining environment itself. In some cases, reminders of the clinical hospital setting acted against patient enjoyment of mealtimes or the ability to socialise [37,54,71]. Furthermore, unlike other institutional dining settings, such as schools, in which sociality can occur through instances such as sharing food with peers [72], hospital patients might have varying nutritional needs and restrictions that are more likely to limit such interactions at mealtimes.

Factors such as these differentiated the experience of communal eating in the hospital from other social dining environments. Despite this, findings from the included studies on the importance of creating a friendly and appealing dining environment align with explorations into the impact ‘servicescape’ has on social behaviour outside of hospitals [73].

In some cases, pushing tables together to facilitate a social dining experience felt crowded [59], while for others, the structure of the rooms themselves had implications for the acoustics of the space, which were seen as a deterrent to the eating experience [53]. One aspect of the physical environment that seemed to facilitate or enhance a positive mealtime environment was the addition of aesthetic dining room objects such as tablecloths or vases with flowers, as well as more functional objects, such as water jugs or special cutlery [35,38,42]. Whilst children were not participants in these studies, it is likely these findings would apply to them too: elsewhere, it has been shown that younger children consider environmental factors labelled as ‘table artefacts’ to play an important role in creating a ‘good’ eating experience in a kindergarten setting [71]. Objects such as flowers, proper crockery, and tablecloths were perceived by children as conducive to a more enjoyable and cosier mealtime, while simultaneously being appraised for their practical functions [71].

In Young et al.’s qualitative study of older inpatients [60], participants argued that the ideal scenario for a hospital dining experience would be to recreate a sense of homeliness, a concept that also arose in Jong et al.’s qualitative study involving older adults [55], where it was reported that eating in a home-like environment made for a more comfortable experience. Xia and McCutcheon also speculate as to how the introduction of a dining table to an acute care facility for older adults might create a more ‘natural’ and homelier mealtime environment instead of the reported silence that existed during mealtimes as patients ate in bed [74].

Studies included in this review also found that visitors played a key role in recreating a sense of home away from home by bringing in food cooked at home and eating with patients, aligning with other key works in this area [75]. Whilst not included in this review, Coyne’s research on children’s experiences of hospitalisation that involved CYP as participants observed that some voiced concerns about being separated from the atmosphere of their home and missed home-cooked meals [13].

It is important to note that, for patients, eating in the hospital is often distinguished from eating in other places by the physical discomfort or transformation that requires them to remain under hospital care. As Sidenvall et al. note, patients might be experiencing pain caused by their treatment or condition and want to focus on eating, rather than socialising at mealtimes [51]. Dornan et al.’s research with patients being treated for head and neck cancer draws on the ‘conscious process’ that eating with others became during treatment [76]. Changes in functional abilities meant patients could no longer enjoy the same foods as their tablemates, both inside and outside of the hospital, and patients became self-conscious of their appearance during mealtimes, leading to feelings of stress. Others have reported on increased experiences of nausea and food aversions related to some treatments [77], such as chemotherapy, and have noted that, in children, such symptoms make eating difficult, especially around other individuals [78]. Elsewhere, Mårtensson et al. report that mealtimes for children who are tube-fed create unfamiliar mealtime situations both for those relying on these feeding methods and for others in the dining environment [79]. Despite this, Dornan et al. observed that patients who were able or motivated to take part in social eating viewed it as something that had a positive effect on well-being, or ‘life satisfaction’ [67].

### 4.1. Strengths and Limitations

This review only included papers written in English, possibly limiting results to studies carried out in particular cultural contexts and excluding others that might have provided more diverse perspectives on social eating, a concept often conceived in various ways across cultures [9]. Future research might include results written in different languages in order to propose guidance that considers a variety of different cultural needs and practices.

The use of only two databases to conduct the search due to restrictions regarding capacity may also have limited results in this area. However, the decision to identify further papers via the bibliographies of the included studies was made to capture as many relevant works as possible.

An advantage of choosing a scoping review is the ability to include a broad range of studies that utilise various approaches, methods, and sample groups, developing a comprehensive account of the type of evidence available in a certain area. It is therefore important to recognise that social eating was characterised and measured in different ways and was observed at different levels across the studies, which is a potential reason for the gaps in robustly collating and presenting this research. Where other types of review, such as systematic reviews, can present synthesised data and thus provide a basis from which practical recommendations may be derived, data collation in scoping reviews cannot be carried out in the same way, and conclusive comparisons are not as easily reached [80]. This review does not provide definitive guidance based on the studies it includes, but instead highlights some initial implications for practice, especially those relevant to children’s eating in the hospital.

Future research might employ a systematic review using a wide range of databases to explore the existing literature in more depth and to offer a synthesised account of the findings that might offer a grounding to make recommendations for practice.

### 4.2. Reflections Related to Children’s Eating in Hospital

While some papers included in this review include a range of participants involved in the mealtime experience and delivery, such as staff and families, future research might build upon aspects of care and considerations that are particular to a paediatric context. Certain paediatric conditions might have particular implications for children’s eating in the hospital that need to be considered. For example, sensitivity to the sensorial aspects of eating with others might need to be considered further for CYP with autism spectrum disorder [81,82] as well as children with cancer [83]. It is also important to recognise the distinct social nature of many children’s lives and the disruption to this that hospitalisation can cause, especially for those with more severe conditions [83]. Others have also reported on the value of social spaces other than dining rooms in hospitals to cater to the social needs of CYP [84].

‘Family meals’ have been a key focus of research, often characterised—and idealised [85]—as convivial occasions, with shared mealtimes upheld in some academic research and public opinion as central to the development and well-being of the child as well as the reproduction of the family unit [22,86,87]. Alongside this body of research, in clinical practice, and especially in the context of paediatric nursing, ‘family centred care’ has become a key tenet that aims to consider the needs and values of, as well as support for, the whole family [88]. Mårtensson et al.’s work, for example, notes that parents experienced feelings of loneliness during mealtimes [46]. Further research in this area might focus on the presence of children’s parents and carers during mealtimes and make recommendations for how social eating might support visitors as well as patients. Though the presence of family members has also been shown to improve the hospital care for other vulnerable patient groups [89], CYP are particularly dependent on the presence of their parents or carers during their time in hospital not only for support regarding their well-being, but also as playing a crucial role in advocating for their child’s needs and autonomy during treatment [90,91].

Furthermore, the reviewed studies found that eating with parents or family members was important in making the eating experience more ordinary [46,62]. In Gibson et al.’s qualitative exploration of paediatric oncology patients spending time in and out of hospital, it was observed that interactions with family and friends at mealtimes improved the children’s food intake, distracting them from feelings of discomfort they experienced when eating [92].

The increase in food intake observed by Gibson et al. is supported by the studies included in this review [92], as nearly all (5/6) of the studies measuring nutritional intake saw a significant increase when patients ate in a social setting during mealtimes, though further studies could contribute to these findings by exploring whether these impacts are found with larger sample sizes. While maintaining adequate levels of nutrition and avoiding malnutrition should be key considerations across all patient groups, these findings are particularly important to consider in the context of CYP. Maintaining adequate nutrition and an optimal weight is an important area of supportive care, especially since infants, children, and adolescents have the extra requirement of growth and development [78] (p. 209). Elsewhere, it has been suggested that adolescents who were overweight were more likely to report eating dinner alone than others, suggesting that eating with others not only affects intake level but also what food is being consumed. Outcomes such as these could impact significant factors such as patient length of stay; future works might build on this research regarding social dining and levels of food intake in the context of a paediatric ward.

## 5. Conclusions

Based on the findings from the studies included in this review, social eating in hospitals has been shown to impact dietary intake and nutritional outcomes and to have implications for patient well-being. These effects are moderated by a range of factors, including the age and needs of the patient group, the eating environment, and the presence of staff and visitors. The studies suggest that social dining in a hospital setting should include environmental and sensory considerations, consider the diversity and complexity of patient needs, and recognise that outcomes of eating with others will depend on who patients are eating with, and the impact of their condition or treatment on the ability to eat. Since the literature also suggests that the role of hospital staff is important in the delivery and facilitation of social dining, the capacity of staff to meaningfully engage in mealtimes should also be considered.

## Appendix A

**Table A1:** Data charting for the studies included in this scoping review.

First Author, Date	Setting	Aim of Study	Study Design	Methodology	Patient Characteristics	Participants	Sample Size	Context	Key Findings Relating to the Research Question
Baptiste 2014 [28]	Ottawa, Canada. Geriatric rehabilitation center containing 60 beds.	To explore geriatric patients’ perceptions regarding eating at the bedside vs the common dining room.	Qualitative	Semi-structured individual interviews.	Aged 65 and over, eight patients, five women and three men.	Patients	8	Bedside eating or common dining room.	>Patients reported limited perceived control in choosing locations (staff). >Mood influenced location, e.g., feeling sad = not wanting to socialise. >Physical abilities, e.g., pain and fatigue, played a major role. >More time to eat in rooms and save personal time. >Loneliness and boredom were experienced when eating in rooms. >Mobility involved in moving to the dining room was seen as a benefit by patients. >The dining room environment was seen as pleasant, e.g., windows. >Possibility to socialise but also highlights tensions, e.g., if people do not talk. >½ said they preferred eating in the dining room, two had no preference, and two said they preferred eating in their rooms.
Beck 2017 [39]	Neurological care unit, Denmark	To investigate how health professionals experience participating in a mealtime intervention inspired by the concept of Protected Mealtimes (P.M) and intended to change mealtime practices.	Qualitative	Three focus groups with five participants in each. Semi-structured based on the mixed ‘funnel’ model.	Patients with varying neurological conditions	15 health professionals working in a neurological ward, with mixed levels of exposure to the ‘Quiet please’ intervention. Mixture of roles, e.g., social worker, secretary, speech therapist.	15	‘Quiet please’, goal = to change mealtime practices by creating an environment inspired by protected mealtimes. Based on PM but modified for the Danish neurological care context. Reduced noise and goal of calmness across the ward.	>Created a sense of togetherness across patients because they had something they could share. >Changes to the environment—e.g., folded napkin, flowers on tables, etc.—meant patients behaved differently, e.g., made their beds.
Beck 2017 [38]	Danish neurological unit	To explore the experiences of patients who were admitted to the neurological ward during an intervention, inspired by Protected Mealtime, that changed the traditional mealtime practice	Qualitative	13 semi-structured interviews were conducted with patients.	Aged 30–83 with neurological conditions	Patients	-	‘Quiet Please’ intervention	According to the patients, being considered as people made them experience the meal as a meal shared with other people and not as an institutional obligation. The aesthetic elements provided the patients with conversation topics that were unrelated to their diagnoses and symptoms. This was essential, according to the patients, because ‘small talk’ during the meal led to relationships with other patients, which potentially helped them eat a little more. Also, there were negative reactions to it, e.g., ‘Quiet Please pisses me off. It is nice to sit and talk or chat like we do at home when we sit and eat’. During the interviews, the aesthetic elements that were provided in the intervention, e.g., flowers on the tables, were shown to be meaningful to the patients because they conveyed a sense of care for the mealtime setting. The patients explained that their conversations with each other during mealtimes were important to develop a feeling of being at home while hospitalized, and mealtimes then became a common topic of conversation.
Beck 2018 [48]	Denmark. Neurological unit with three small wards	To study what patients who are afflicted with a neurological disease experience and assign meaning when participating in mealtimes during hospitalisation.	Qualitative	10 semi-structured interviews.	10 participants aged between 27 and 78 years with neurological diagnoses.	Patients	10	Mealtimes were eaten either at tables in the hallway, in bed, or at shared tables next to the bed.	>Many chose to eat in their beds to maintain privacy; the hallway setting was stressful, and it was not possible for patients to eat with visitors due to the hectic and busy hallway dining area. >Equally missed social interaction. >Socialisation was sometimes made tricky by the varied conditions patients had; the bed was a safe place. >Conditions and treatment sometimes made eating unappetizing.
Beck 2019 [57]	Danish neurological unit	To examine the meaningfulness of the phenomenon of hospital meals for hospitalized patients with a neurological disease.	Qualitative	23 interviews.	Patients with varying neurological conditions	23 participants with different neurological diseases/diagnoses. Aged 27–83.	23	Mealtimes on a neurological disease unit	>Patients explained that they often felt lonely during mealtimes. >Found a community with fellow patients through identifying each other’s loneliness during mealtime situations, which helped facilitate a sense of normality. >Eating in bed is associated with calmness and comfort. >Patients described how just sitting together and eating (even without long conversations) could be a peaceful activity, a positive association with the possibility of small talk that avoided serious thoughts about their current condition. Though staff did not eat with patients, it offered opportunities to become acquainted with them.
Bryon 2008 [50]	Geriatric–psychiatric ward in a Belgian Academic Hospital. Long-term treatment ward for older people	To obtain insight into the care process surrounding mealtimes within a geriatric–psychiatric ward from the perspective of the caregivers	Qualitative	Participant observation, semi-structured interviews, and focus groups.	Patients had long-existing psychiatric (schizophrenia, personality disorder, etc.) and/or physical problems (diabetes, Parkinson’s disease)	Head nurse, five nurses, three nursing aides, a worker, the psychologist, the occupational therapist, and the nurse manager.	13	Meals were served in two common dining rooms: one for residents who eat normal food and one for residents on a special diet. Those needing eating assistance shared a table. A total of 34 residents were spread over two dining rooms, eating with three to four persons at a table. The dining rooms were small, which led to problems with wheelchairs. In a rather limited space, a large number of residents were present, which resulted in a lot of commotion.	Caregivers experience that with their help, the mealtime can become a moment of (a) social interaction, (b) attention to self-care, and (c) enjoyment. By eating in the common dining room, residents are taken out of their isolation. Moreover, residents stimulate and supervise each other to leave their rooms. During the meals, social skills can be practiced and stimulated. Eating together makes the mealtime more enjoyable and promotes appetite. Patients also laid the table and put their plates away, giving them agency. Agreements were made with patients about eating etiquette in the dining room; if not followed, then they would eat in their rooms. Individualizing the meal in accordance with the different needs and wills of patients is difficult when using this form of mealtime care. Patients who lived in a closed ward would eat together even when at times, some would probably want to eat alone. However, allowing some patients to eat alone or letting them choose dining location makes it harder for staff to manage. Made the dining room look nice, e.g., using decorations and special menus, etc., during holidays. Those with special diets ate in a separate dining room, as otherwise, patients traded their meals and took things from the plates of others; it was not easy to work in this kind of dining room.
Davies 1980 [34]	Liverpool, UK The dayrooms of two wards in a continuing-care geriatric hospital.	To provide greater opportunities for social interaction and for choice over the way the meal is served.	Quantitative	Participant observations and any social interactions were recorded.	Patients were of similar medical status, all had multiple handicaps, and nearly all were confined to wheelchairs.	27 patients (7 men) in Ward A and 25 women in Ward B	27	In Ward A, most patients sat with their backs to the walls, and one row of wheelchairs was lined across the centre of the room. Ward B had a smaller dayroom. Three groups of patients sat at round tables, the others in rather cramped conditions around the walls. The trolleys had to be kept in the corridor. Intervention for making Ward A more social changed the seating such that there were two tables, each seating six, and introduced decorations, such as a set of cloths, water jugs, etc. Trolleys were moved to the corridor to reduce noise.	Ward B had more interactions and more varied interactions at the beginning of the study. After the intervention, there was an increase in social interactions in Ward B. Three factors deserve consideration: the ‘social distance’ between staff and residents, the physical environment, and the role of the ward’s sister. When placed at tables, talk was facilitated, and helpful behaviour became possible.
Dickinson 2005 [44]	26 bed units in the UK.	To implement patient-focused mealtime practice for older patients within a hospital unit and promote healthy ageing by improving mealtime care by working towards a patient-focused and enabling culture.	Qualitative	Focus groups, interviews, and observations of six mealtimes.	Older patients with complex discharge needs	19 staff took part in focus groups, interviews with six patients, and observations of ward mealtimes.	25	The physical environment was poor, with plastic garden tables used in the dining room as tables.	>Social aspects considered poor by staff and patients affected by the environment, but also from a lack of facilitation by staff. >Occurred only by chance. >All patients interviewed said they would like the opportunity to share their meals with staff from the unit; one patient had performed this before and said it was beneficial.
Edwards 2004 [31]	Women’s Health Unit in a National Health Service (NHS) hospital.	To ascertain how food intake might be affected by allowing hospital patients to eat in the company of others	Quantitative	Plates were weighed after patients had eaten, and nutritional analysis was carried out.	Patients were hospitalised for a variety of surgical procedures in the age range of 36–89.	Patients at table (4) aged 36–62 years, with a mean age of 49. Patients by the bed (5) aged 60–86 years, with a mean age of 75. Patients in bed (4) aged 49–89 years, with a mean age of 63. This was only a small cohort.	4	Group 1. Around a table Group 2. Sitting by their bed Group 3. Sitting in bed	>Significant increase (*p* < 0.05) in mean daily energy intake for the group sitting around the table (Group 1) over the other two groups. >When individual meals are compared, there were no significant differences in the groups for the evening meal; at the midday meal, Group 1 was significantly different from both Groups 2 and 3; and at breakfast, the only significant difference was between Groups 1 and 2
Furness. 2023 [53]	All sites of Austin Health, a teaching hospital in Australia. Including a large tertiary acute hospital (Austin Hospital, AH), a subacute, aged-care and rehabilitation setting (Heidelberg Repatriation Hospital), and a dedicated rehabilitation setting (Royal Talbot Rehabilitation Centre)	To describe the mealtime experience using the qualitative components of the Austin Health Patient Mealtime Experience Tool (AHPMET) to complement the quantitative findings of this tool.	Mixed methods	Questionnaire with opportunities for participants to provide qualitative feedback.	Adult inpatients. The median age was 77 (range 19–101) years, and the median length of stay was 19 (range 1–270) days.	149 inpatients who had received meals for at least one full day.	149	The majority (75%) of participants consumed their meals in an individual or shared room, while the remainder (25%) consumed their meals in a shared dining room	>Environment theme identified factors that inhibit mealtime intake and are a deterrent to the mealtime environment, including sensory aspects of the environment, other patients, accessibility and functionality of the mealtime setup and dining area, and clinical impact symptoms. ‘The structure of the room makes it really noisy and loud—the high ceilings and big openness echoes noises—loud chattering and loud banging and crashing of the dishes in the kitchen and plates being cleared—makes it hard to hear, not peaceful’ (male, 40 years, LOS 165 days). >Facilitators to mealtime/nutritional/food intake and a pleasant MTE were also identified, such as socialisation during meals and a mealtime environment that facilitates such interaction. Additionally, the available furniture used during mealtimes affected intake and/or comfort. ‘Was able to choose who I got to eat with and talk which was good’ (male, 77 years, LOS 13 days). >Atmosphere theme had 9 positive comments and 22 negative ones (perceived negatively) >Features of the physical environment, including noise, visitors and other patients, room surroundings and ambience, interruptions by hospital staff, and smells or odours were not commonly reported to affect food intake in the study.
Gardner 2022 [41]	Cotswold House ED unit in Oxford, UK	To understand the system of our dining room, including the environment, purpose, processes, and how people interact with these and each other	Mixed methods	Observations in the dining room and recording the number of eating disorders, and qualitative feedback gathered from patients and staff about the intervention.	The average age of patients admitted is 28.9 (17.1–60), 97% identify as female.	Patients and staff	-	Three changes were introduced, including (1) a host role in the dining room, (2) a guide to the dining room for new staff along with competencies, and (3) a dining goals group. Staff normally eat with patients in the dining room; however, because of infection control changes through the pandemic, staff have been unable to eat with patients, which has led to the loss of role modelling of normal eating in the dining room.	The dining room has been reported by patients and staff as a very stressful environment. For patients, this can manifest itself in increased eating disorder behaviours such as eating very small mouthfuls, hiding food, smearing food, or eating foods in a certain order, which leads to maintaining the eating disorder. Results to date show a 33.44% reduction in observed ED behaviours between baseline and the post-test period.
Gardner 2021 [40]	Cotswold House ED unit in Oxford, UK	To decrease the number of ED behaviours at mealtimes in the dining room through the implementation of initiatives identified through diagnostic work.	Mixed methods	A survey was conducted to assess mealtime protocols across 22 eating disorder units, and semi-structured interviews were conducted with staff at three units.	On average, six patients were in the dining room at mealtimes. All patients admitted in 2019 were women and ranged from 19 years to 68 years old (the mean age is 33 years). The average length of stay across 2019 was 73 days. Most had anorexia nervosa.	Patients and staff	-	Mealtimes occurred six times a day in the ED wards. An introduction to the host’s role was performed. During the pandemic, staff stopped eating with patients. Two dining rooms were in the unit; the main dining room was a small room with four tables and was for those in recovery, while the upstairs dining room was for those meeting criteria.	Reduction in the number of ED behaviours observed could be that the dining room feels less chaotic and more predictable as a result of the host role, which has addressed ED behaviours triggered by anxiety and distress from environmental disturbances. The most frequently observed behaviours were unusual eating behaviours during mealtimes, for example, tearing up food, being detached at mealtimes/not talking or making conversation, and becoming anxious about unexpected changes to meal service. Patient feedback themed around feeling more supported by staff and the dining room feeling more organised. Both staff and patients acknowledge that mealtimes in the dining room are still a difficult experience, but much can be achieved.
Hartwell 2013 [35]	UK, two orthopaedic wards in an acute care hospital	To evaluate the attitudes of staff towards implementing a dining room eating experience in a hospital ward by considering not only physical constructs but also the social domain and operational practice of staff providing the hospitality.	Qualitative	Interviews with hospital staff.	Patients were undergoing elective surgery for hip/knee replacements and had a length of stay of approximately 10 days. 12 males, 6 females.	Staff involved in group dining were interviewed.	18	Enhanced dining environment facilitating the creation of communitesque experiences. The tables were covered with a tablecloth and laid with cutlery, crockery, glasses, jugs of water, and condiments. This environment was well lit and provided a quieter atmosphere than normally found in wards.	Easier to serve food, e.g., courses, food stayed hot. Reduced the amount of legwork needed by staff with everything in one place. Provision of a more ‘dignified’ and ‘civilised’ environment in which patients could eat. Providing a group dining experience encouraged patients to become mobile and increase their motivation. Patients enjoyed the social experience of the meal. More hygienic- away from the clutter of beds. Gave a sense of ‘normality’, recreating mealtimes that they may have at home while providing an environment that is familiar. Support/clinical staff observed that patients at the very early stages of recovery were likely to be in too much pain to want to socialise. Some patients may still require catheters and drips, and in those circumstances, feel that to be wheeled into a dining area is too undignified, inappropriate, and uncomfortable.
Hartwell 2016 [64]	Acute Care Hospital with 26 wards including medical, elective surgery, maternity, and intensive care (UK/NHS)	To identify and examine all perceived aspects of the meal experience from the patient’s viewpoint and to quantify the impact of each one	Quantitative	Questionnaire with scalable answers drawn up from qualitative interviews.	The mean age was 69.1, with the minimum being 25 and the maximum being 94 years old. Orthopedic ward patients.	296 responses, 120 males, 176 females, with 2 individuals saying they had never eaten hospital food before	296	Eating at the bedside	>On social, staff, and ward, the more experienced respondents tended to be more positive, although differences here were not statistically significant (*p* > 0.05). >Demonstrated from first principles that food quality, followed by service quality, were the most important predictors of customer satisfaction, thereby confirming findings of some previous authors. After this, the social environment, the personal characteristics of the patient, and the immediate eating environment were the most important factors.
Holst 2017 [36]	Three departments in Aalborg University Hospital, Denmark, with around 26 beds.	To improve energy and protein intake by improving the aesthetics of the surrounding environment and providing individualized meal services.	Mixed methods	Observational study, 24-h food intake registrations for 3 d consecutively, a questionnaire, and a semistructured patient interview.	Patients from three departments: (medical infectious diseases, haematology, and heart–lung surgery)	30 patients with a mean age of 62.9. Mean LOS-6 days before intervention. A total of 37 patients with a mean age of 67.2 after intervention	30	Changes to the environment—table cloths and small vases—were purchased. Coloured tray mats and napkins were introduced for all main meals. Lastly, soothing background music was played during lunch and dinner. Also, nutritional information was given to patients.	>Patients found the environment very welcoming and inviting, allowing patients to socialize more with each other during meals. One patient made the following comment: “The atmosphere is company increases appetite, eating more with others”. >Overall group showed significant improvements in energy intake
Jong 2021 [55]	Australia Subacute care wards 1x rehabilitation ward and 1x geriatric evaluation and management ward	To understand and explore staff’s perspectives and experiences of communal dining in subacute care, and the impacts on staff mealtime practice	Qualitative	Participant observation and ethnographic/semi-structured interviews.	Older patients from a geriatric and rehabilitation ward.	Staff involved in nutrition care or present on the ward at mealtimes. Broad range of professionals included.	-	>The rehabilitation ward had a small dining room at the rear. >The geriatric ward had a large, central dining room.	>Three themes identified: (i) benefits to patients, (ii) logistical and practical challenges, (iii) supportive cultural factors. >Change of scenery, home-like environment, more comfortable and easier transition. >Staff played a role in higher levels of socialisation. >Positive relationship with intake and mobilisation. >Transportation and time pressures are difficult; the location of the dining room relates to levels of supervision. >Dining room design impacts convenience, e.g., toilet locations and table designs not being wheelchair friendly can impede socialisation. >Cognition impacts the desire to use the dining room. >Staff encouragement and normalisation of the dining room have an impact on use.
Jonsson 2021 [59]	Sweden, four wards within two Swedish public hospitals that care for adult medical, orthopaedic, and geriatric patients	To explore how hospitality was performed by nursing staff and meal hosts in the dining room environments at four hospital wards, and to explore the specific role of the room and its artefacts in facilitating or hindering acts of hospitality	Qualitative	Ethnographic study, non-participant observations with interactions initiated by staff or relatives (researcher not involved in provisioning).	-	Nursing staff, staff involved in preparing food, and relatives of patients. The patients who were present in the dining room environments were indirectly observed. However, the focus was not on what the patients did or said but on how the staff acted towards the patients.	-	Both wards A and B had different dining room settings. A—Dayrooms with two tables and six assigned seats, used for dining and other activities. B—Five or six tables with six chairs at each table, chairs have wheels, and meal boxes on display in fridges.	The ability to perform hospitality during mealtimes differed between the wards and could be hindered as well as facilitated by the location of the dining room environment and the materiality within. Hospital A, silence in the room was emphasised by the noise of hospital machinery. In hospital B, the function of a meal host was observed to promote a sense of commensality for the patients during mealtimes. The patients were not alone, even if they dined alone at their tables. During one observation at hospital A, the dining room was reorganized to enable nine patients to sit together and eat their lunch. The tables were brought together, creating one long table. This arrangement seemed to facilitate and promote a positive atmosphere for the patients during the meal, as well as ensuring that several nursing staff attended the meal service. However, rearranging the tables also created a crowded feeling in the room.
Justesen 2014 [56]	The gynaecology and cardiology wards of a Danish Public Hospital in the eastern part of Denmark	To introduce and explore whether the application of the participant-driven photo-elicitation (PDPE) research method in a hospital meal context can contribute to a richer insight and understanding of the experiences and perceptions of hospital meals. It aims to expand the conceptualisation of hospital meals by providing access to a multi-sensory response to meal experiences.	Qualitative	Photo elicitation and follow-up interviews.	Patients from the gynaecology ward and the cardiology ward	Eight patient participants, aged 19–81. Four males, four females	8	Buffet trolley system onwards	>Patients ate alone. However, opportunities for social activity took place around the buffet trolley. Patients could relate to each other and created a patient community. >Some patients avoid social interactions while eating to maintain sense of identity.
Justesen 2016 [65]	The gynecology and cardiology wards of a Danish Public Hospital in the eastern part of Denmark	To explore how hospitality can be co-created in a hospital food environment and how it emerges from socio-material interactions	Qualitative	Ethnographic, structured, and unstructured observations carried out over 6 months. Interviews based on photo elicitation.	Patients at the gynecology ward were mainly cancer patients or patients hospitalized for surgery.	-	-	Dinner served from a buffet trolley at lunch onwards.	>Patients themselves also took the initiative to transform the hospital room into a hospitality space, e.g., pushing chairs against a dinner table and calling it a ‘cafe’, also others commented on how the physical space made a difference—e.g., eating in a chair more.
Larsen 2021 [54]	Denmark. North Denmark regional hospital, and Aalborg University Hospital	To identify the experiences of patients about eating situations, wishes, and needs in connection with meals during their stay in the hospital.	Qualitative	20 semi-structured interviews that lasted between 7 and 54 min	-	Aged 51–92. Twenty participants chosen based on sex, age, and surgical and medical departments to capture the nuances of the patient experience.	20	At both hospitals, patients could eat food in the living room or in their wards. At Aalborg, Healthcare Professionals (HCPs) presented patients with a menu and served the requested food. At North Denmark Hospital, patients who were able to walk around could choose food from the buffet.	>The hospital setting affects the eating experience, does not create a meal break, and is reminiscent of illness. >Eating around others might serve as a motivation to eat more. >Eating together can provide an opportunity for company and conversation. >Physical discomforts influence how they experienced the meal, e.g., vomiting/spilling food. >Own weaknesses or disabilities affected their desire to eat in front of others, as well as their dignity. >Social interaction depends on who they are eating with.
Long 2012 [52]	UK, three NHS and one independent eating disorder service, all four sites included inpatient care and meals as part of that treatment.	To investigate inpatient perceptions of mealtimes on eating disorder units.	Qualitative	Individual semi-structured interviews with participants.	Patients from one independent unit and three NHS units.	12 patients, 5 from an independent unit and 7 from the three NHS units. All were females, with a mean age of 22 years.	12	Group meals in the dining room for a period of over two weeks	>Dining room organisation/layout is important. For example, table shapes, sizes, and distances from one another create different dining experiences. >Distractions. For example, staff talking and the radio on can be both helpful and a hindrance. >Emotional experiences. For example, being new to the ward, feelings of anxiety, embarrassment, and panic. >Behaviours learnt from other patients and ‘feeling sucked into the way people are eating’, comparisons made with other patients. >Rivalry between patients. For example, who took the longest time to finish the meal, feelings of being watched and judged. >Loss of identity through lack of individualisation.
Long 2012 [58]	UK, 22 eating disorder units, children, adolescents, as well as adults. In total, 14 (63.6%) were NHS services and 7 (31.8%) were independent units (one unit did not provide this information). Five (22.7%) units provided care for those under 18 years old, 10 (45.5%) for over 18, and 7 (31.8%) for all ages.	To increase our understanding of the way in which mealtimes are currently conducted within specialist eating disorder units, and second, to qualitatively explore staff perspectives of mealtimes.	Mixed methods	Survey questionnaire and follow-up interviews with staff from selected units.	The units catered for a range of ages, from under 18 (beginning at 11 years old) through to adult units	Individual interviews were conducted with 16 staff members (2 males and 14 females) who had varying lengths of experience in providing inpatient care	16	The three ED units they used for interviews all had dining rooms.	Thirteen units (59%) reported that ward staff would at least sometimes sit with patients without themselves eating a meal. Reasons for this included unit policy not to eat with patients and personal choice (such as having plans to eat following their shift). The majority of units (90.9%) reported at least sometimes having non-nursing members of staff eating with patients. Creating a calm dining environment was important in reducing mealtime stress. Staff found meals daunting and emphasised how they felt watched while eating, as if they were punishing people. Staff opinions were split as to whether they should be expected to eat alongside patients or not. Many staff believed this to be an important factor for patients, as it provided role models and normality for the situation. Others saw having to eat alongside patients as distracting from the care they provided. Some staff felt they should eat with patients, but chose not to, because they did not feel comfortable.
Markovski 2017 [29]	Australia, subacute setting across two Western Health Care services	To investigate the effect of the ‘Dining with Friends’ programme on energy and protein intake in hospitalised elderly patients, identify whether patient groups at risk of malnutrition could benefit from a communal dining environment, and identify patients’ preferred environment for meal consumption.	Quantitative	Data collected regarding food intake and patient satisfaction on 54 separate midday meals. Used a malnutrition screening tool (MST)	Majority in rehabilitation for 1–2 weeks and had a cognitive impairment. The mean age was 79 years., 73% were female, 45% were screened as being at risk of malnutrition, and 24% reported a poor appetite.	34 patients. Excluded patients who needed feeding assistance or where it was not deemed socially appropriate for them to participate	34	Comparison between dining room and bedside meal experience. Two midday meals observed, one in the bedroom and one in the dining room.	>Intake of protein and energy increased by 20% when the meal was consumed in the dining room. >Majority of patients identified the dining room as their preferred eating site, 68%. >All groups identified at risk of malnutrition consumed more energy and protein in the dining room. The intake of energy and protein increased by 30% when patients who were underweight (BMI < 22) ate in the dining room. The intake of energy and protein increased by 30% when patients identified with significant cognitive impairment ate in the dining room.
Mårtensson 2021	Paediatric care facility in Sweden	To investigate whether the Five Aspect Meal Model could be appropriate for children with a gastrostomy tube in caring science and paediatric care.	Qualitative	Semi-structured interviews	Children being treated for cancer, using gastrostomy tubes	Patients and their families/parents, three children and four parents included.	7	Mealtimes for children with gastrostomy tubes in hospital and at home. Families of children.	>Flexibility needed as mealtimes occurred in different areas/rooms, e.g., dining table, bed, and couch. >Kitchen environment experienced as warm, personal, and relaxed, and described as an optimal place for mealtimes. >Issues eating orally meant patients did not always want to eat at the table. >Room environment had an impact on the desire to eat/appetite. >Most mealtimes spent in bed, even when the family was eating at the table. >Educational meals seen as more social and enjoyable by parents. >Parent loneliness during mealtimes.
Mathiesen 2021	Denmark. Specialized acquired-brain injury unit.	To identify and resolve issues in the existing acoustic environment of a common dining area of a hospital ward. Explore how improvements to the acoustic eating environment, including music playback, affect patients’ mealtime experience, behaviour, and food intake. Examine various musical genres and their appropriateness for eating situations in hospital settings.	Mixed methods	Plates were weighed after patients finished each meal throughout each phase. Participant observation, social interactions, and comments about the intervention were captured (1 = no interaction, 5 = lots of interaction). Semi-structured face-to-face interviews were carried out with patients.	17 patients with an acquired brain injury: 11 males, 6 females. The mean age was 64.5 years.	Patients	17	Common dining room for patients. Soundproofing materials were installed in Phase 2 In Phase 3, music was introduced to the common dining area.	The amount of social interaction decreased from the Baseline (Mean Rank = 99.79) to Phase 2 (Mean Rank = 81.85) and subsequently increased in Phase 3 (Mean Rank = 83.62), H2 = 8.745, *p* = 0.013. All patient interactions were, however, reported as being positive in the observation form, and no observations of agitated behaviour were made at any point throughout the study. The concept of commensality saturated the interviews and was articulated as a significant and indispensable part of the participants’ daily lives (inside and outside the hospital). Prior to the acoustic intervention, hospital noises were perceived as getting in the way of being able to talk to one another; intervention meant they were able to talk to one another with more ease. Cosiness helped interpersonal relationships. The content of conversations shifted away from serious topics and created commonalities between people. Increased enjoyment from food. Average fluid intake increased.
Melin 1981 [33]	Sweden. Ulleraker hospital.	To evaluate the effects of changes in furniture arrangements and mealtime routines on two types of behaviour- communication and appropriate eating	Quantitative	Observations of communication occurring at mealtimes between patients and between patients and staff	Psychogeriatric patients.	21 patients, 15 diagnosed with senile dementia, 2 suffering from cerebral atherosclerosis, 2 with presenile dementia, and 2 with chronic schizophrenia. The mean age was around 81 years.	21	Ward contained four-bed rooms, two-bed rooms, and single-bed rooms, as well as a lounge, a dining area, and an occupational therapy room. One experimental group and one control group. Changes in the physical environment were introduced in the second week, and changes to mealtime routines were introduced in the third week for the experimental group. Ward was sparsely decorated, and furniture was placed along the walls. Patients in the experimental group were placed around small tables and, instead of trays, were given saucers, cups, etc. Patients were able to serve themselves, with staff not present, during coffee time. At mealtimes, patients grouped around small tables with serving dishes, salt, pepper, napkins, soft drinks, beer, etc.	>Significant increase in communication in the experimental group. >Inactivity might have been influenced by the social setting. >The changed meal situation meant that patients had to communicate to acquire what they wanted from the table.
Neo 2021 [62]	One of the largest tertiary hospitals in Singapore. Eight medical and four geriatric wards.	To explore enrolled nurses’ perceptions of providing nutritional care to hospitalised older people in Singapore’s acute care setting.	Qualitative	Descriptive study. Individual face-to-face semi-structured interviews.	Older patients who required more assistance in nutritional care	15 enrolled nurses aged between 26 and 49	15	Patients eating in bed/at the bedside	Participants felt that families’ involvement was critical in providing nutritional care to hospitalised older people; they recommended that families actively participate in hospital mealtimes to improve their nutritional intake. For example, families bringing food and eating together recreate a home environment and encourage patients to eat.
Ottrey 2018 [61]	One subacute ward from each of two locations within a large metropolitan healthcare network in Australia	To explore multiple perspectives and experiences of volunteer and visitor involvement and interactions at hospital mealtimes. In addition, to understand how the volunteer and visitor role at mealtimes is perceived within the hospital system.	Qualitative	75 ethnographic and semi-structured interviews and participant observation	Patients were typically admitted to these wards for geriatric care or rehabilitation, for example, after a stroke or fracture.	45 staff (including six leaders), five volunteers, and 11 visitors.	61	Meals eaten in bed with the option of dining room	Visitors were observed to facilitate shared dining experiences, often eating their food while sitting with the patient at mealtimes, enriching their experience of being in the hospital, and bringing in food. Nurses described how volunteers assisted at mealtimes by helping patients if needed and supervising those eating their meals in the dining room. Visitors and volunteers were seen as helping with well-being and providing interaction at mealtimes, although not all ate with the patient.
Paquet 2008 [43]	Rehabilitation unit of a university geriatric facility in Eastern Canada	Part of a broader investigation whose aim was to study the psychological and organizational determinants of food intake in institutionalized elderly patients. To build upon the interpersonal circumplex model of human interactions to evaluate how specific elements of the meal social environment contribute to the social facilitation of elderly patients’ intake.	Quantitative	Mealtime interactions observed and assessed for meal intake based on the proportion left on the plate.	Patients with a 4-week average length of stay. Over 65 years old.	32 patients	32	Common dining room with a capacity of 24 patients	>The total number of interactions observed for participants and their interaction partners was positively related to energy intake. >Food intake by participants was associated with their communal behaviors, but not with their agentic behaviors. >Interaction per se may not be enough to explain the impact of the social environment on intake, and the specific nature of these individual behaviors and their complementarity may play an important role in the effect. The communal behaviors expressed by these patients had a positive impact on the amount of energy consumed by participants for that meal.
Rosbergen 2019 [32]	Regional hospital, Australian 16-bed acute stroke unit.	To explore the effect of environmental enrichment within an acute stroke unit on how and when patients undertake activities, and the amount of staff assistance provided, compared with a control environment (no enrichment)	Quantitative	Measuring the proportion of time doing physical, cognitive, and social activities	Stroke severity ranging from mild to severe among older adults.	A total of 30 participants in the control group and 30 in the enriched group. Mean age of control group = 76.0 and mean age of enriched group = 76.7. Control group: 56.7% male and 43.3% female. Enriched group: 73.3% male and 26.7% female.	60	The control group received standard therapy and nursing care that was provided mainly at the participants’ bedside. Embedding environmental enrichment included the transformation of public spaces in the acute stroke unit to communal seating areas for patients and families. Stimulating equipment (such as iPads loaded with therapy apps, music, books, newspapers, art, games, puzzles, and magazines) was distributed throughout communal areas and at the patient’s bedside, accessible 24 h a day, with communal breakfast and lunch also included.	>Environmental enrichment increased physical and cognitive activity and reduced time spent in bed on weekends. >Scheduled communal activity and provision of stimulating resources within a clinical environmental enrichment significantly contributed to increased activity levels in stroke survivors.
Sidenvall 1996 [51]	Rehabilitation and long-term care clinic with four wards, Sweden	To investigate cultural values and ideas concerning table manners and food habits expressed by patients in geriatric care. Studying the elderly’s perceptions of food habits in contrast to food served in the common dining room.	Qualitative	Informal ethnographic interviews comparing habits at home to those in the dining room	Stroke, fracture, rheumatoid arthritis, Parkinson’s disease, and peripheral circulation insufficiency were represented, even though the patients had several other diagnoses.	23 females, 19 males. Born and raised in Sweden. Geriatric, with a mean age of 81	42	The patients had their meals in a common dining room.	Patients with the retained ability to eat reported discomfort and loss of appetite due to the inability of others to remain clean. Such situations occurred when table-mates were unable to separate their own things from collective bowls, used their own spoon in the jam pot, or dug for cubes in the sugar basin with their fingers. ‘Troublesome atmosphere caused by eating problems that arose from conditions or treatment’. Difficulties in socialising when in pain or discomfort and wanting to focus on the food. Patients without handicaps seeking social contact found it difficult to initiate a conversation with handicapped table-mates or with those who had impaired hearing, bad sight, or were confused. Also expressed negative emotions, e.g., ‘sorrow’ when seeing that fellow patients were declining; their appetites becoming worse. On the other hand, healthy patients also expressed satisfaction with their table-mates and found the meal situation to be a moment of fellowship.
Sidenvall 1994 [42]	Two rehabilitation and long-term care Wards in Sweden	To investigate individual patients’ meals in geriatric care with respect to both the intentions of the nursing staff and assessments of patients, as well as to those patients’ experiences and the extent to which they expected to be able to influence the meal situation regarding behaviour and table manners, eating competence, and diet.	Qualitative	Ethnographic interviews, observations, and recorded data.	Dependent patients = 3 and independent patients = 15. Diagnoses varied from stroke to Parkinson’s to hip fracture.	18 patients, 13 females and 5 males, with a mean age of 81. They were mixed with patients who were not allocated to the study in the dining room. 21 enrolled nurses, all female and with a mean age of 41, also took part.	39	Common dining room, patients divided in the room between those with dependent and independent abilities to eat. Patients also divided into two groups depending on eating ability, need for assistance, and conduct in the dining area.	Both patients and nursing staff strove to create a meal situation that was as natural and independent as possible. Measures taken to ensure collective dining and independent eating, e.g., cups and special cutlery. One participant did not want to eat in the dining area and had to be pushed to. After exposure to the dining area, she seemed satisfied. One patient had reacted to the table manners of a fellow patient. Some patients helped others at the table. Those with more severe physical conditions felt that they could not reach their own standards of behaviour at the table and found it difficult to eat in the common dining room. Found more freedom eating privately. Those with moderate eating problems all wanted to eat in the dining room. Despite some silence at the table, patients felt an affinity with others. Some reported unpleasant behaviours at the table. Those able to eat with ease said that their experiences depended to a great extent on the behaviour of others, which varied from positive to negative.
Sidenvall 1999 [49]	Rehabilitation and long-term care clinic with four wards, Sweden	To examine and explain the institutional organization of meals, drawing on Goffman’s theory of institutionalized culture, Elias’s theory of the ‘civilising process’, Douglas’s theory of purity and order, and Bourdieu’s key concept ‘habitus’.	Qualitative	Informal ethnographic interviews and participant observations	Older patients with varying conditions	1st period: 13 elderly women, 5 elderly men, and their respective personal nurses 2nd period: 23 elderly women and 19 elderly men were interviewed twice. A total of 7 registered nurses and 17 enrolled nurses interviewed. The mean age of the patients studied during the first research period was 81.5 years; during the second period, 76.5 years	84	In the dining room, patients moved when nurses decided it would be better if they were in a more isolated area of the room or their own rooms—e.g., if they ate with their fingers or shouted or swore. Nurses wanted to create a homelike atmosphere.	Almost none of the caregivers working in the dining room asked the patients about their experiences of being there. Those who failed to eat according to their standards, i.e., their civilized manners, which before hospitalization was their habitus, felt shame and kept silent about their failure.
Sundal 2020 [62]	Norwegian general paediatric unit	To explore the experiences of parents and nurses and the concrete ways in which nurses and parents collaborate in partnership when caring for hospitalized preschool children	Qualitative	Participant observation and interviews	The children were in the beginning stages of hospitalization, probably staying for 2 days or more. They were neither critically nor terminally ill, and were between 1 and 6 years old. Eight girls and three boys with various medical diagnoses, four of whom had chronic medical conditions (2 × 1-year-old, 4 × 2-year-old, 4 × 3-year-old, and 1 × 6-year-old).	12 parents (3 fathers and 9 mothers) of 11 hospitalised children and 17 female nurses participated.	29	Optional dining room/bedside eating	>Talks about a mother and child both eating at a table together. >Staff perspectives around the impact of parents on the child’s wellness and eating behaviour. >Parents play an important role in making the meal familiar to the child.
Sundal 2023 [47]	A paediatric unit in a Norwegian hospital	To investigate how parents and nurses experience collaborating and sharing responsibilities and tasks when providing home-like care for hospitalized children in everyday situations	Qualitative	Participant observation	Children between the ages of 1 and 6 years with various medical diagnoses	Twelve parents of eleven hospitalized children and 17 nurses who cared for the children	29	Parents and families given the option of eating in the dining room together or in their own rooms.	-Option of eating together in their own dining room created an idea of homeliness and familiarity. -One child did not want to eat in bed and wanted to eat at the table.
Walton 2013 [45]	Aged care rehabilitation centres in three Australian hospitals	To (1) describe ward activities which have a positive or negative influence on dietary intakes, (2) determine the times taken to start and complete meals, and (3) make recommendations that would make the ward environment more conducive to eating at mealtimes.	Mixed methods	Interview administered questionnaires and participant observation	Observations: 14 male and 16 female patients were observed, and their activities documented. The mean age was 79.2 years. Interviews: 11 patients	Patients, 10 nurses, and 1 doctor	41	Bedside meals vs dining rooms. Two hospitals had dining rooms, and the other did not.	>The primary eating location was the bedside. However, when available, a dining room was very popular for mobile patients at lunch and teatime. >Improved socialisation between patients and staff was certainly observed in this study at the two hospitals, which had a dining room. >The private hospital provided patients with their meals one course at a time, with all plate covers removed, and plates from earlier courses were cleared as they were finished. The dietary intakes seemed higher for some patients in the private hospital dining room. The social approach to the meal, the number of decanted food and beverage items, the ambience of the setting, and the additional mealtime assistance afforded by the private hospital were certainly conducive to enhanced mealtime enjoyment and dietary intakes. >Positive interruptions included social interaction, which improved consumption. >Makes recommendations for the use of the dining room.
Wright 2006 [30]	Charing Cross Hospital, London. Elderly acute wards	To investigate the effect of eating in a supervised dining room on nutritional intake and weight, for elderly patients in the acute medicine for the elderly ward.	Quantitative	Food intake and weight data were collected over the study period for each patient.	30 patients attended the ward dining room at lunchtime; 18 patients acted as the control group, eating at their bedside. The median age was 84 years, and there was no significant difference for age, gender, diagnosis, or initial weight between the control and dining room groups. Each patient visited the dining room a median of four times (interquartile range: 2–7).	Patients	30	The dining room was established in one ward, and patients were encouraged to attend every lunchtime during weekdays. Patients in the second ward only ate at their bedside and acted as a control group.	>Dining room group had significantly higher intakes of energy than the control group. >Mean energy intake from the lunch meal for the dining group was 489 kcal (438–554), and the mean energy intake for the control group was 360 kcal (289–448). >No significant increase in weight gain or protein intake. imitation: The median number of times visiting the dining room was 4.
Young 2024 [60]	Five acute care wards in a metropolitan teaching hospital in Brisbane, Australia (general and renal medicine; general urological and vascular surgery)	To gather and understand the experience of hospital mealtimes from the perspectives of those receiving and delivering mealtime care (older inpatients, caregivers, and staff) using photovoice methods to identify touchpoints and themes to inform the co-design of new mealtime interventions	Qualitative	Photo-voice method	Older patients with varying conditions	Older inpatients, caregivers, and staff directly involved in mealtime care. Overall, 21 participants (10 patients, 5 caregivers, and 6 staff) took part in observations, and 13 participated in interviews (4 patients, 3 caregivers, and 6 staff)	21	Eating usually in bed or at the bedside	>Ideal scenario—the environment would be as homely as possible, with the patient tables being clean and cleared of clutter, and the patient would be sitting in a chair. >All agreed that the ideal mealtime would involve and welcome families and caregivers, with caregivers appreciative of also being provided with a meal, conviviality = ideal, reality = isolation

**Table A2:** Search terms used to carry out the database search in Scopus. Asterisks (*) are used in this table to indicate multiple characters or ‘wildcard’ searches.

Population	(child*) OR (adolescent*) OR (“young W/3 people”) OR (“young W/3 person”) OR (teen*) OR (“young W/3 adult”) OR (“young W/3 adults”) OR (kids) OR (youth) OR (famil*) OR (parent*) OR (“school W/3 age”) OR (minor*) OR (“patient*”) OR (“inpatient*”)
Concept	((“hospital”) OR (“hospital W/3 ward”) OR (“medical W/3 ward”) OR (“pediatric W/3 unit”) OR (“paediatric W/3 unit”) OR (“hospital W/3 care”) OR (“subacute W/3 care”) OR (“acute W/3 care”) OR (“oncology W/3 unit”) OR (“children’s W/3 ward”) OR (“pediatric W/3 care”) OR (“paediatric W/3 care”) OR (“young people’s W/3 ward”) OR (“young person’s W/3 ward”) OR (“ward”) OR (“hospital W/3 unit”) OR (“pediatric W/3 ward”) OR (“ENT W/3 unit”) OR (“gastroenterology W/3 unit”) OR (“surgery W/3 ward”) OR (“orthopaedic W/3 unit”) OR (“orthopedic W/3 unit”) OR (“physiotheraphy W/3 unit”) OR (“physiotheraphy W/3 ward”))
Context	((“social W/3 eating”) OR (“social W/3 dining”) OR (“social W/3 tables”) OR (“social W/3 table”) OR (“communal W/3 dining”) OR (“collective W/3 dining”) OR (“eating W/3 together”) OR (“dining W/3 together”) OR (“sharing W/3 food”) OR (“shared W/3 mealtimes”) OR (“shared W/3 mealtime”) OR (“sociable W/3 eating”) OR (“sociable W/3 mealtimes”) OR (“sociable W/3 mealtime”) OR (“family-style W/3 meals”) OR (“family-style W/3 meal”) OR (“eating W/3 alone”) OR (“solitary W/3 eating”) OR (“solitary W/3 dining”) OR (“commensal W/3 eating”) OR (mealtime*) OR (commensa*) OR (“communal W/3 table”) OR (“family-style W/3 dining”) OR (“group W/3 dining”) OR (“food W/3 service”) OR (“eating W/3 environment”) OR (“eating W/3 location”))
